# The Mechanism of Ferroptosis and Applications in Tumor Treatment

**DOI:** 10.3389/fphar.2020.01061

**Published:** 2020-07-22

**Authors:** Xinyue Lin, Jieyi Ping, Yi Wen, Yan Wu

**Affiliations:** ^1^ School of Medicine, Jiangsu University, Zhenjiang, China; ^2^ Gynecology, The Fourth Afﬁliated Hospital of Jiangsu University, Zhenjiang, China

**Keywords:** ferroptosis, mechanism, cancer, drug, drug resistance

## Abstract

Iron-dependent ferroptosis is a new form of cell death in recent years, which is driven by lipid peroxidation. The lethal lipid accumulation caused by glutathione depletion or inactivation of glutathione peroxidase 4 (GPX4) is characteristic of the ferroptosis process. In recent years, with the in-depth study of ferroptosis, various types of diseases have been reported to be related to ferroptosis. In other words, ferroptosis, which has attracted widespread attention in the fields of biochemistry, oncology, and especially materials science, can undoubtedly provide a new way for patients. This review introduces the relevant mechanisms of ferroptosis, the relationship between ferroptosis and various cancers, as well as the application of ferroptosis in tumor treatment. We also sorted out the genes and drugs that regulate ferroptosis. Moreover, small molecule compound-induced ferroptosis has a strong inhibitory effect on tumor growth in a drug-resistant environment, which can enhance the sensitivity of chemotherapeutic drugs, suggesting that ferroptosis is very important in the treatment of tumor drug resistance, but the details are still unclear. How to use ferroptosis to fight cancer, and how to prevent drug-resistant tumor cells have become the focus and direction of research. At the end of the article, some existing problems related to ferroptosis are summarized for future research.

## Introduction

Ferroptosis was first described by Dr. Brent R. Stockwell in 2012, and then gradually entered the research boom of most researchers. In 2018, ferroptosis was set by the Nomenclature Committee on Cell Death (NCCD) as a form of regulated cell death (RCD) (Galluzzi et al., 2018). Supported by experimental data, it is not difficult to find that ferroptosis and other types of cell death are very different in morphology, genetics, and biochemistry. Ferroptosis breaks the fact that almost all RCD in mammals is caused by the activation of caspase-dependent apoptosis (Fuchs and Steller, 2011). Besides, the particular cell necrosis pattern of ferroptosis is reduced mitochondrial volume, increased mitochondrial membrane density, and decreased mitochondrial cristae (Dixon et al., 2012). This is very different from the observation results of apoptosis and necrosis by electron microscope. Generally, ferroptosis can be inhibited by iron chelators and lipophilic antioxidants, and inhibitors of apoptosis, autophagy, and necrosis such as caspase inhibitors and necrostatins (Berghe et al., 2010) cannot inhibit ferroptosis (Lei et al., 2019). In the absence of key effectors such as BAX, BAK, MLKL, and RIPK1/3, ferroptosis will still occur. However, there are some connections between these cell deaths. Studies have found that ferroptosis can promote renal tubular necrosis in the kidney disease model (Linkermann et al., 2014). At the same time preventing ferroptosis and necrosis may bring benefits to delay the development of the disease. Tumor suppressor p53 has been involved in the research of apoptosis in the past, but the research of p53 in ferroptosis has also gradually increased (Jiang et al., 2015b).

Overall, ferroptosis is an RCD that is dependent on the content of iron and lipid hydroperoxide. A large number of lipids accumulate in the cells, disrupting the balance of the redox reaction and ultimately leading to cell death.

## Mechanism

**Figure 1:**
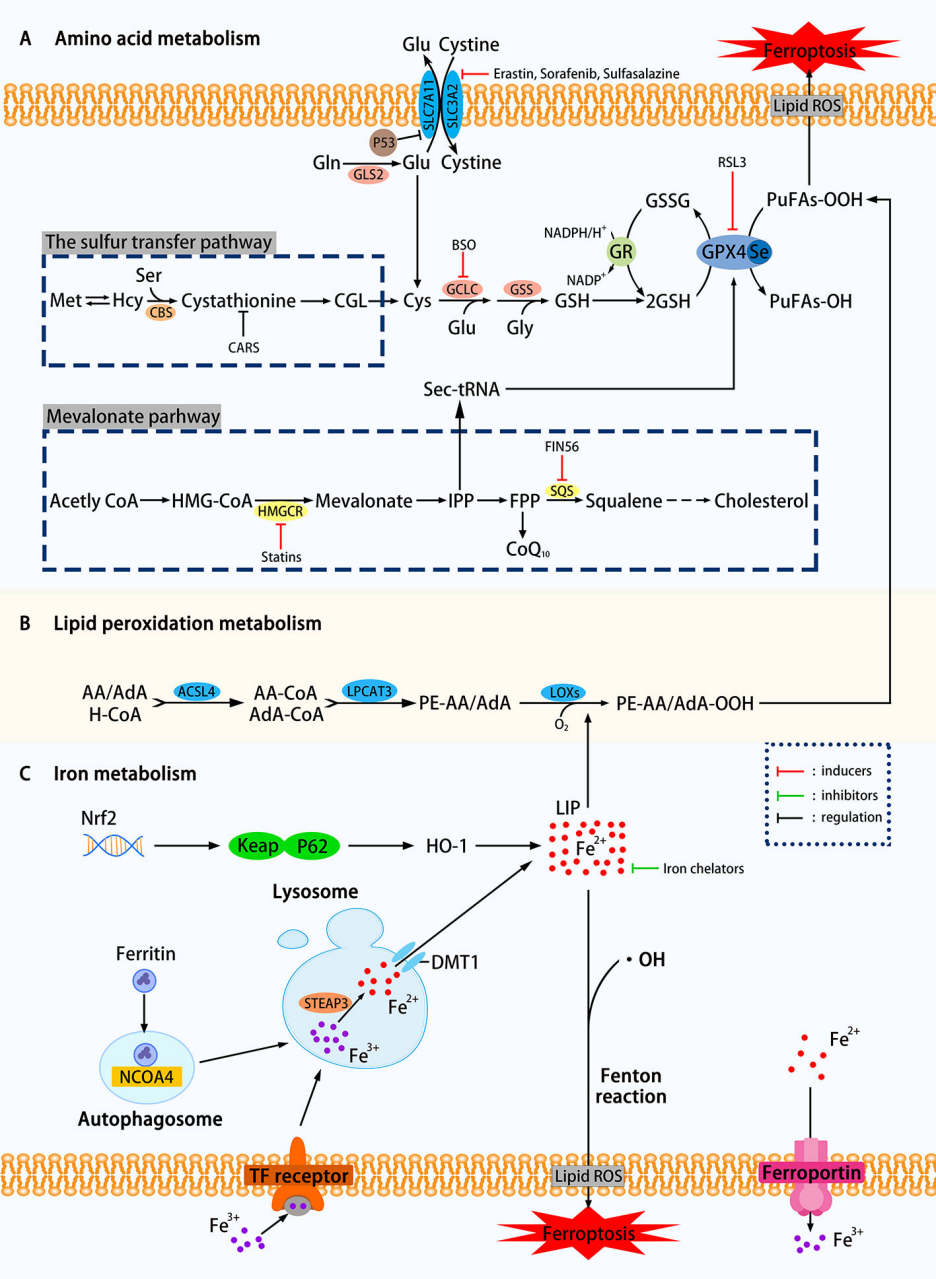
Mechanisms of ferroptosis in a cell. **(A)** The regulation of amino acid metabolism can be divided into glutathione consumption and reduced activity or abundance of GPX4. The decomposition of glutamine, the availability of cysteine is reduced, and the high concentration of glutamic acid outside the cell can affect the synthesis of glutathione and Eventually trigger ferroptosis through a series of effects. Methionine uses the transsulfation pathway to synthesize cysteine to avoid Xc-system effects that can affect ferroptosis. The sec-tRNA and CoQ10 produced in the mevalonate pathway can also affect ferroptosis. **(B)** Lipid metabolism can play a role in ferroptosis through both enzymatic and nonenzymatic pathways. **(C)** Ferritin autophagy, iron input and output, etc. can use Fenton reaction or Fenton-like reaction to trigger lipid peroxidation and ferroptosis. Transcription factor Nrf2 is also involved (SLC7A11, the glutamate/cystine antiporter solute carrier family 7 member 11; SLC3A2, the glutamate/cystine antiporter solute carrier family 3 member 2; GSL2, glutaminase 2; GOT1, glutamic-oxaloacetic transaminase 1; GSH, glutathione; CBS, cystathionine *β*-synthase; GCLC, glutamate-cysteine ligase; GSS, glutathione synthetase; GR, glutathione reductase; GPX4, glutathione peroxidases 4; RSL3, Ras-selective lethal small molecule 3; ACSL4, acyl-CoA synthetase long-chain family member 4; LPCAT3, lysophosphatidylcholine acyltransferase 3; LOXs, lipoxygenases; PUFAs, polyunsaturated fatty acids; LIP, labile iron pool; DMT1, divalent metal transporter 1; HMGCR, HMG-CoA reductase; SQS, squalene synthase; IPP, isopentenyl pyrophosphate; ROS, reactive oxygen species; NCOA4. nuclear receptor coactivator 4; FIN56. ferroptosis-inducing agents 56.)

From the above definition, there are two keywords for the primary mechanism of ferroptosis: iron dependence and lipid hydroperoxide accumulation. In this review, we divide the mechanisms that affect ferroptosis into amino acid metabolism, lipid peroxidation metabolism, iron metabolism, etc. The related mechanism of ferroptosis is shown in **Figure 1**.

### Glutathione Consumption

As shown in **Figure 1**, using the glutamate-cystine reverse transport system, also known as Xc-system, intracellular glutamate (Glu) replaces extracellular cystine in a 1:1 manner, which can be converted to cysteine and used for the synthesis of glutathione (GSH). GSH, as an intracellular antioxidant buffer, maintains redox balance *in vivo* under the action of GPX4. We divide the upstream factors that affect GSH consumption into three small points: decomposition of glutamine (Gln), decreased availability of cysteine and high extracellular glutamate.

#### Decomposition of Glutamine

Over 60% of the free amino acids in the human body are in the form of glutamine in muscles and other tissues. The high extracellular concentration of glutamine is converted to Glu under the catalysis of glutaminase (GLS1 and GLS2). Glutamate uses the deamination reaction to form α-ketoglutarate (α-KG), which is then degraded by the mitochondrial tricarboxylic acid cycle (TCA). Knockdown of GLS2, which is the inhibition of glutamine decomposition pathway, can inhibit ferroptosis has been experimentally proven (Gao et al., 2019). Inhibition of aminotransferase GOT1 by amino-oxyacetate (AOA) can inhibit ferroptosis in mouse embryonic fibroblasts (MEFs) (Villar et al., 2015).

#### Decreased Availability of Cysteine

**Figure 1** clearly showed that the decline in cysteine availability caused by various causes has strongly promoted the occurrence of ferroptosis. In general, cancer cells can resist increased ROS by up-regulating the glutamate/cystine antiportersolute carrier family 7 member 11 (*SLC7A11*) and avoid cell death caused by oxidative stress. Research by Jiang et al. confirmed that *TP53* could transcriptionally inhibit *SLC7A11*, prevent cystine uptake, reduce the availability of cysteine, and increase the sensitivity of H1299 cells to ferroptosis (Jiang et al., 2015a). Yet many things have their twists, and some cells can use methionine to synthesize cysteine by the sulfur transfer pathway, avoiding ferroptosis induced by Xc-system inhibitors such as Erastin (a Ras-selective lethal compound) (Stockwell et al., 2017). The cysteinyl-tRNA synthetase that uses cysteine for protein translation of tRNAs is encoded by the *CARS* gene. Hayano et al. found that three additional Ambion Silencer Select siRNA sequences targeting the *CARS* demonstrate the ability of the *CARS* knockout to prevent Erastin-induced ferroptosis, which is related to the increase of intracellular free cysteine (Hayano et al., 2016). The cystathionine *β*-synthase (CBS) can convert homocysteine to cystathionine, which can be converted into cysteine by the corresponding cystathionine *γ*-lyase in the sulfur transfer pathway. To study the effect of the sulfur transfer pathway on drug resistance, the authors knocked out both *CARS* and *CBS*, the latter inhibited the conversion of homocysteine to cysteine (inhibition of the sulfur transfer pathway), and found that compared with *CARS* knockout cells alone, HT-1080 are resensitive to Erastin treatment (Hayano et al., 2016). This study showed that upregulation of the sulfur transfer pathway may help restore the sensitivity of drug-resistant cells to ferroptosis. However, this pathway acts on the "upstream" of ferroptosis, so it cannot prevent ferroptosis induced by the GPX4 inhibitor RLS3.

#### High Extracellular Glutamate

It can be seen from the synthetic pathway of GSH that the level of Glu inside and outside the cell is also an influencing factor of ferroptosis. When the extracellular Glu concentration is at an abnormally high level, the equal exchange of Glu and cystine is affected, which indirectly affects the import of cysteine, and eventually leads to ferroptosis due to the accumulation of lipid peroxidation. In various neural cell injury models, Glu and its analogs can induce experimental animals to develop a brain injury syndrome similar to seizures (Liu et al., 2017), which is likely to support further the above view (Olney, 1990).

Overall, any of the three changes based on excessive glutamine breakdown, decreased cysteine availability, and high extracellular Glu concentrations can silence the Xc-system, causing GSH-depleting ferroptosis. The conversion from cysteine to GSH requires the catalysis of two enzymes: glutamate-cysteine ligase (GCLC) (Griffith, 1999) and glutathione synthetase (GSS) (Dickinson and Forman, 2002). GSH is an intracellular antioxidant buffer substance. Sun et al. were the first to analyze the relationship between ferroptosis and GSH depletion, concluded that the consumption of GSH leads to the accumulation of lipid peroxidation in retinal pigment epithelial (RPE) cells, triggering ferroptosis (Sun et al., 2018). Glutathione can be considered an essential point in a series of reactions to ferroptosis.

### Decreased Activity or Abundance of GPX4

GPX4, responsible for the reduction of lipid hydroperoxides (PUFAs-OOH) to lipid alcohols (PUFAs-OH) (Lv et al., 2019), is a selenocysteine-containing enzyme, one of eight glutathione peroxidases (GPX1-GPX8) (Brigelius-Flohe and Maiorino, 2013). It uses two molecules of GSH as a donor to reduce PUFAs-OOH to the corresponding alcohol. The by-product GSSG is reduced to GSH using Glutathione reductase (GR) and NADPH/H^+^. When GPX4 is affected, it will directly increase the oxidative stress, and then ferroptosis will follow. The current programs that use GPX4 to induce ferroptosis include reducing the activity or abundance of GPX4, etc. Ras-selective lethal small molecule 3 (RSL3) was originally thought to alter iron-related proteins and genes that caused the accumulation of polyunsaturated fatty acids and then caused cell death. Still, after a series of complex experiments, it was found that its mechanism is to directly silence GPX4 to increase oxidative stress to trigger ferroptosis (Yang et al., 2014). K. Shimada et al. found through experiments that the mechanism by which ferroptosis-inducing agents 56 (FIN56) works is to reduce GPX4 abundance by consuming GPX4 protein (Shimada et al., 2016). Some other genes involved in amino acid metabolism that regulate ferroptosis are listed in **Table 1**. If GSH metabolism is a critical point in the amino acid metabolism mechanism of ferroptosis, GPX4 is the bridge that carries all these changes.

**Table 1:** Genes involved in ferroptosis amino acid metabolism.

Gene	Related protein	Regulation	Effect on ferroptosis	Reference
*SLC7A11*	Solute carrier family 7 member 11	Knockdown: prevent cystine uptake	Promote	(Jiang et al., 2015a; Dixon et al., 2012)
*CARS*	Cysteinyl-tRNA synthetase	Knockout: promote the sulfur transfer pathway	Inhibit	(Hayano et al., 2016)
*CBS*	Cystathionine β-synthase	Knockout: inhibit the conversion of homocysteine to cysteine	Promote	(Hayano et al., 2016)
*GLS2*	Glutaminase 2	Knockdown: inhibit the conversion of glutamine to glutamate	Inhibit	(Gao et al., 2015)
*GOT1*	Glutamic-oxaloacetic transaminase 1	Knockdown: inhibit the synthesis of α-KG	Inhibit	(Gao et al., 2015)
*GCLC*	Glutamate-cysteine ligase	Knockdown: prevent GSH biosynthesis	Promote	(Yang et al., 2014)

### Lipid Peroxidation Metabolism 

It is currently believed that both spontaneous oxidation and enzymatic oxidation are involved in the formation of lipid peroxides.

#### Nonenzymatic Lipid Peroxidation

Spontaneous oxidation, the process of oxygen-dependent free radical chain reaction (formula I-III), is usually divided into three stages: Initiation (I), Propagation (II), termination (III) (Frank, 1950). Initiation (I) usually starts with the generation of a free radical such as ·OH that is sufficiently reactive. When the lipid molecule LH is pumped away by a hydrogen atom, the initial lipid radical L· can be generated. L· continues to propagate (II) through addition, hydrogen pumping, fracture, etc. and repeats to form a chain reaction. As long as the reaction has been dominant, the oxidation process will not stop. Of course, with only a small amount of antioxidants that capture and scavenge free radicals, the reaction can be slowed down or terminated (III). PLOO·and PLO· produced during the spontaneous oxidation of lipid peroxidation are also continuously using adjacent lipid molecules to participate in the propagation and termination of free radical chain reaction (Davies and Guo, 2014). H. J. H. Fenton first described the Fenton chemistry or Fenton reaction (formula IV) in 1894 (Dunford, 2002). It is currently believed that the Fenton reaction, or Fenton-like reaction, in a sense also provides a source of free radicals for lipid peroxidation (Lai and Piette, 1978). In conclusion, the effect of nonenzymatic lipid peroxidation on ferroptosis cannot be ignored.

(I) $$\text{LH}+•\text{OH}\to \text{L}•+{\text{H}}_{2}\text{O}$$

(II) $$\begin{array}{c} \text{L}•+{\text{O}}_{2}\to \text{LOO}•\\ \text{LOO}•+\text{LH}\to \text{LOOH}+\text{L}• \end{array}$$

(III) L•+L•→L−L

(IV) $$\begin{array}{c} \text{F}{\text{e}}^{2+}+{\text{H}}_{2}{\text{O}}_{2}=\text{F}{\text{e}}^{3+}+•\text{OH}+\text{H}{\text{O}}^{-}\\ \text{F}{\text{e}}^{3+}+{\text{H}}_{2}{\text{O}}_{2}=\text{F}{\text{e}}^{2+}+•\text{OOH}+{\text{H}}^{+} \end{array}$$

#### Enzymatic Lipid Peroxidation

The research focus in enzymatic lipid peroxidation is mainly on lipoxygenases (LOXs) rather than cyclooxygenases (Yang et al., 2016). Mammal LOXs are iron-containing nonheme dioxygenases, which can promote the dioxygenation of free and esterified PUFAs (Kuhn et al., 2015). Yang et al. performed pharmacological inhibition of some ALOX subtypes under GSH depletion conditions, proving that LOXs can affect Erastin-induced cell death, supporting the view that LOXs exerts an effect on ferroptosis. The metabolism of PUFAs involves two more important enzymes, namely, acyl-CoA synthetase long-chain family member 4 (ACSL4) and lysophosphatidylcholine acyltransferase 3 (LPCAT3). The latter has weaker resistance to ferroptosis than the former (Doll et al., 2017). PUFA-CoA is formed by polyunsaturated fatty acid (PUFA) catalyzed by acyl-CoA synthase. Arachidonic acid (AA) is an ω-6 polyunsaturated fatty acid, and is usually preferentially thioesterified by ACSL4 and incorporated into phospholipids (Golej et al., 2011). Phospholipids oxidize to form phosphatidy-lethanolamine to drive ferroptosis in cells. Doll et al. observed that the production and spread of PUFAs-OOH were blocked by knocking out the *ACSL4* gene or pharmacologically inhibiting ACSL4 with thiazolidinediones, and ferroptosis was effectively suppressed. It is worth noting that, compared with the difficulty of growing cells without Gpx4 (Seiler et al., 2008), cells that knock out both genes of *ACSL4* and *GPX4* can grow normally in cell culture, which makes us pay attention to the coordination between GPX4 and ACSL4 effect (Doll et al., 2017). In addition, when PUFA-PEs hydroperoxide derivatives are added to inactivated GPX4 cells, the sensitivity to ferroptosis is higher (Kagan et al., 2017). Yang et al. discovered through lipidomics that PUFAs, which contain easy-to-extract diallyl hydrogen atoms, are most susceptible to peroxide during ferroptosis, and preventing this peroxidation by adding deuterated PUFAs that are not easily oxidized on the diallyl carbon to the cells can inhibit ferroptosis (Yang et al., 2016). In some cell lines, lipid ROS can activate the mitogen-activated protein kinase (MAPK) pathway (eg ASK1-p38/JNK pathway) (Nakamura et al., 2019) to induce ferroptosis (Yu et al., 2015). Ye et al. found that knockdown *Ras* in HL-60/NRAS^Q61L^ cells can reduce JNK/p38 phosphorylation, knockingdown *Ras* or pharmacologically inhibiting the JNK/P38 pathway (SP600125/SB202190) can cause Erastin-induced increase in TfR1 expression and induce ferroptosis (Ye et al., 2019). Other genes involved in lipid metabolism that regulate ferroptosis are listed in **Table 2**.

**Table 2:** Genes involved in ferroptosis lipid metabolism.

**Gene**	**Related protein**	**Regulation**	**Effect on ferroptosis**	**Reference**
*ACSL4*	acyl-CoA synthetase long-chain family member 4	Knockout: prevent PUFAs-COA formation	Inhibit	(Kagan et al., 2017)
*Lpcat3*	lysophosphatidylcholine acyltransferase 3	Knockout: prevent PUFAs-COA from being incorporated into phospholipids	Inhibit	(Kagan et al., 2017)
*Gpx4*	glutathione peroxidase 4	Knockout: prevent the reduction of lipid hydroperoxides (PUFAs-OOH) to lipid alcohols (PUFAs-OH)	Promote	(Yang et al., 2014)
*LOX*	Lipoxygenases (lipoxygenase-12/15)	Knockout: prevent the dioxygenation of free and esterified PUFAs	Inhibit	(Yang et al., 2016)
*AKR1C*	aldo-keto reductase family 1 member C1	Upregulation: promote the elimination of end products of lipid peroxidation	Inhibit	(Dixon et al., 2014)
*ACSF2*	acyl-CoA synthetase family member 2	Knockdown: prevent the formation of specific lipid precursors in mitochondrial fatty acid metabolism.	Inhibit	(Dixon et al., 2012)
*CS*	Citrate synthase	Knockdown: prevent the formation of specific lipid precursors in mitochondrial fatty acid metabolism.	Inhibit	(Dixon et al., 2012)
*SQS*	squalene synthase	Knockdown: prevent the depletion of antioxidant CoQ10	Inhibit	(Shimada et al., 2016)

At present, the exact mechanism from lipid peroxidation to ferroptosis in cells is not clear. Some researchers believe that this may be related to the formation of micelles and pores in the membrane (Borst et al., 2000). Agmon et al. used molecular dynamics models to analyze the characteristics of lipid bilayers during ferroptosis, and found that lipid peroxidation affects the permeability, fluidity, and curvature of the membrane, so that the formation of micelles and pores on the membrane triggers cell death (Agmon et al., 2018). Some scholars also believe that PUFAs metabolism is associated with the production of toxic secondary products such as 4-hydroxynonenal (4-HNE) (Zhong and Yin, 2015).

### Iron Metabolism

Iron is one of the very few and indispensable trace elements in the human body. The human body has a perfect mechanism for the regulation of various proteins and pathways to ensure that the iron maintains a balanced state in both the cell and the whole. IRP1 and IRP2 are iron regulatory proteins that can regulate iron metabolism genes such as *TFRC, FTH1*, etc. to maintain the stability of labile iron pools (LIP, composed of a small amount of free Fe^2+^) in cells. Iron homeostasis plays a significant role in normal cell survival and development, while iron accumulation is one of the signs of ferroptosis. As a carrier protein of serum iron, transferrin is endocytosed into cells under the action of the transferrin receptor (TFRC). Transferrin and TFRC are considered to be essential regulators of ferroptosis (Gao et al., 2015), therefore, upregulating TFRC to enrich the cellular iron pool (increasing unstable iron intake) can regulate ferroptosis (Yang and Stockwell, 2008). Besides, by regulating the iron load of ferritin, it also has a specific effect on ferroptosis. Nuclear receptor coactivator 4 (NCOA4) is a crucial receptor for ferritin autophagy (ferritin phagocytosis) under iron homeostasis. It can accumulate ferritin and release iron through lysosomal degradation. The depletion of NCOA4 will prevent lysosomal localization of ferritin and reduce sensitivity to ferroptosis, researched by Mancias et al. (2014). The metal reductase STEAP3 is for converting Fe^3+^ to Fe^2+^. Fe^2+^ in lysosome is released into cytoplasmic LIP through divalent metal transporter 1 (DMT1). Knocking down FANCD2 can inhibit the expression of STEAP3 mRNA in bone marrow stromal cells (BMSCs) induced by Erastin (Song et al., 2016). The lethal effect of labile iron pools may be explained by Fenton chemistry and iron-dependent enzymes (such as lipoxygenase) mentioned earlier. By providing small amounts of antioxidants and iron chelators, the spread of this iron-dependent lipid peroxidation can be prevented. Genes involved in iron metabolism that regulate ferroptosis are listed in **Table 3**. Unlike the ferroptosis inducers described previously, FINO_2_ (an endoperoxide-containing 1,2-dioxolane) does not inhibit the Xc-system and does not act directly on GPX4 like Erastin and RSL3. FINO_2_ has the dual effects of iron oxidation and indirect inhibition of GPX4 enzyme activity, which can induce ferroptosis (Gaschler et al., 2018).

**Table 3:** Genes involved in ferroptosis iron metabolism.

**Gene**	**Related protein**	**Regulation**	**Effect on ferroptosis**	**Reference**
*TFRC* (*TfR1*)	Transferrin receptor	Upregulation: increase unstable iron intake	Promote	(Yang and Stockwell, 2008)
*NCOA4*	Nuclear receptor coactivator 4	Knockdown: prevent degradation of ferritin in the autophagy pathway	Inhibit	(Hou et al., 2016; Mancias et al., 2014)
*IREB2*	iron response element-binding protein 2	Knockdown: regulate the translation and stability of iron consumption-related mRNAs	Inhibit	(Dixon et al., 2012)
*ATG13*	Autophagy related protein 13	Knockdown: prevent degradation of ferritin in the autophagy pathway	Inhibit	(Gao et al., 2016)
*FTH1*	Ferritin heavy chain 1	Knockdown: reduce major iron storage proteins in cells	Promote	(Yang and Stockwell, 2008)
*HMOX1*	heme oxygenase 1	Upregulation: promote heme degradation and free iron release	Promote	(Kwon et al., 2015)
*PHKG2*	phosphorylase kinase, γ2	Knockdown: indirectly promote iron depletion	Inhibit	(Yang et al., 2016)
*HSPB1*	heat shock protein beta-1	Knockdown: increase iron absorption	Promote	(Sun et al., 2015)
*FANCD2*	Fanconi anemia complementation group D2	Knockdown: increase iron absorption	Promote	(Song et al., 2016)
*CISD1*	CDGSH iron sulfur domain 1	Knockdown: inhibit mitochondrial iron uptake	Promote	(Yuan et al., 2016)
*Fpn*	ferroportin	Upregulation: promote the export of iron	Inhibit	(Zhao et al., 2018)

### Other Mechanisms

In addition to the sulfur transfer pathway (the use of methionine to synthesize cysteine through the sulfur transfer pathway to avoid Xc-system effects), the pathways that affect the sensitivity of ferroptosis include the organelle mediated pathways, Nrf2 pathway, *TP53* pathway, mevalonate (MVA) pathway, FSP1-NAD(P)H-CoQ10 pathway, and so on.

#### Organelle-Mediated Pathways

**Figure 2:**
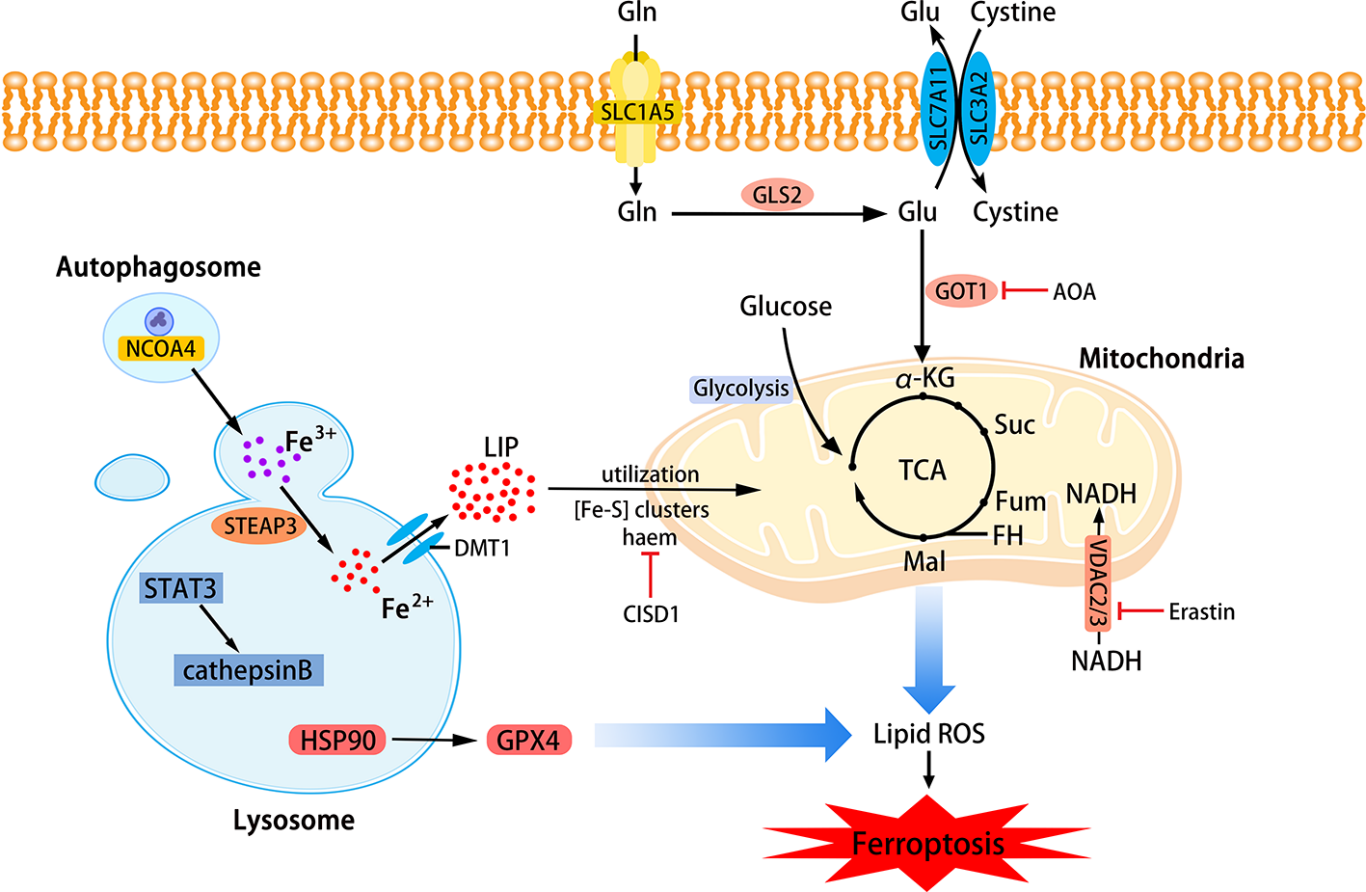
The role of mitochondria and lysosomes in ferroptosis. Inhibition of glutamine decomposition pathway and internalization of mitochondrial Fe^2+^ reflect the indispensability of mitochondria in ferroptosis. Autophagy-dependent ferroptosis also occurs in lysosomes, which involves the participation of multiple proteins. (SLC7A11, the glutamate/cystine antiporter solute carrier family 7 member 11; SLC3A2, the glutamate/cystine antiporter solute carrier family 3 member 2; GSL2, glutaminase 2; GOT1, glutamic-oxaloacetic transaminase 1; α-KG, α-ketoglutarate; TCA, tricarboxylic acid cycle; AOA, amino-oxyacetate; LIP, labile iron pool; CISD1, CDGSH iron sulfur domain 1; ROS, reactive oxygen species; NCOA4, nuclear receptor coactivator 4; HSP90, heat shock protein 90; GPX4, glutathione peroxidases 4; VADC, voltage-dependent anion channel.)

Phospholipids that can be affected during ferroptosis are spread across multiple cell structures, such as mitochondria, endoplasmic reticulu (ER) lysosomes, etc. (Feng and Stockwell, 2018). The relationship between mitochondria, lysosomes and ferroptosis is shown in **Figure 2**.

##### Mitochondria

Mitochondria are the main place to maintain the energy supply of cells and aerobic respiration. Characteristic changes in mitochondria in ferroptosis include mitochondrial rupture, mitochondrial membrane density, and decreased mitochondrial ridges. The metabolism of glutamine mainly depends on the decomposition of glutamine and the mitochondrial TCA cycle. Without glutamine, Erastin-induced cysteine-deprived ferroptosis will be inhibited (Gao et al., 2015). Transaminase inhibitor AOA is to suppress ferroptosis by inhibiting the glutamine decomposition pathway. Other metabolites of the mitochondrial TCA cycle such as succinate (Suc), fumarate (Fum), and malate (Mal) can all participate in ferroptosis along the path of glutamine decomposition. Fumarase, also known as Fumarate hydratase (FH), is a mitochondrial tumor suppressor (Alam et al., 2005). The loss of FH function will enhance the resistance of cancer cells to ferroptosis. Gao et al. believe that enhanced ferroptosis can promote the suppression of tumors by FH. This also shows that the application of ferroptosis in the field of cancer treatment is meaningful. Gao et al. also found that mitochondrial respiratory chain inhibitors did not prevent the death of GPX4-knockout HT1080 cells. Therefore, mitochondria have an indispensable role in ferroptosis, and its influence is ranked upstream of GPX4 (Gao et al., 2019). Free Fe^2+^ is usually incorporated into haem and [Fe-S] clusters (ISCs) to participate in lipid peroxidation in mitochondria. Mitochondrial outer membrane protein CDGSH iron sulfur domain 1 (CISD1) as an iron sulfur protein can inhibit iron transport in the above process. Inhibition of CISD1 by RNAi technology or pioglitazone pharmacology can inhibit mitochondrial iron uptake to prevent lipid peroxidation and ferroptosis (Yuan et al., 2016). Although mitochondria play a central role in oxidative metabolism, its role in ferroptosis requires deeper research.

##### Lysosome

The role of lysosomes in ferroptosis is also important. Fluorescent ROS sensor detected that lysosomes were the major source of cellular peroxidation of ferroptosis induced by Erastin or RSL3 in human HT1080 fibrosarcoma cells. Although ferroptosis is different from other cell deaths, research on its relationship with other cell death types has been ongoing. Recent research suggests that ferroptosis is an autophagy-dependent cell death, which just proves that lysosomes are closely related to ferroptosis. As mentioned before, the transfer of ferritin to lysosomes by cargo receptor NCOA4 is also an important part of ferroptosis (Mancias et al., 2014). Similar to the effect of *NCOA4* knockout, the knockout of autophagy-related genes *Atg5* and *Atg7* also limits ferroptotic cell death induced by Erastin (Hou et al., 2016). Gao et al. demonstrated that the increase in cathepsin B mediated by *STAT3* in Pancreatic ductal adenocarcinoma (PDAC) cells could trigger lysosomal cell death and enhance the sensitivity of ferroptosis (Gao et al., 2018). The activation of STAT3 requires the participation of the MAPK/ERK pathway. Some researchers have identified 2-amino-5-chloro-N, 3-dimethylbenzamide (CDDO) as a new ferroptosis inhibitor and it is also a necroptosis inhibitor. CDDO can target chaperone heat shock protein 90 (HSP90) to regulate chaperone-mediated autophagy (CMA), thereby preventing GPX4 degradation and ferroptosis (Liu et al., 2020).

##### Endoplasmic Reticulum

In addition to supplying its own needs, lipids synthesized by the ER also supply other membrane cell structures such as golgi, lysosomes, etc. The upregulation of ER oxidative stress markers ATF4 (activating transcription factor 4), *CHAC1* (Cation transport regulator homolog 1) and phosphorylation of eIF2α can be observed during ferroptosis (Dixon et al., 2014). But for now, the relationship between ER and ferroptosis is still elusive.

#### Nrf2 Pathway

The transcription factor nuclear factor erythroid 2-related factor 2 (Nrf2) regulates multiple genes, many of which are associated with ferroptosis, including amino acid metabolism-related genes such as *GCLM, GSS, SLC7A11*, iron metabolism-related genes such as *MT1G, TFRC*, etc. (Abdalkader et al., 2018). Silence against Nrf2 can play a role in resisting ferroptosis in cancer cells.

#### *TP53* Pathway

*TP53* (p53 genes) is a kind of human tumor suppressor gene. The upregulation of GLS2 (instead of GLS1), which is one of the transcription targets for *TP53*, leads to p53-dependent ferroptosis (Jennis and Kung, 2016). Hu et al. found that the expression of wild-type p53 protein largely induced GLS2 mRNA levels in V138/H1299 cells (up to 35 times) cultured at 32° (Hu et al., 2010). *TP53* inhibition of SLC7A11 can also trigger ferroptosis (Jiang et al., 2015a). In colorectal cancer, *TP53* also shows its dual induction of ferroptosis (Xie et al., 2017).

#### MVA Pathway

Selenocysteine-tRNA (Sec-tRNA) uses the direct product of the MVA pathway, isopentenyl pyrophosphate (IPP), to promote the maturation of GPX4 (Warner and Berry, 2000). Statins inhibiting HMG-CoA reductase (HMGCR) down-regulates the MVA pathway and reduces the production of IPP and antioxidant CoQ10 (Viswanathan et al., 2017), which is thought to exacerbate ferroptosis. FIN56 is thought to use either degradation of GPX4 to induce ferroptosis, or activation of squalene synthase (SQS) to promote depletion of CoQ10 to induce ferroptosis (Shimada et al., 2016).

#### FSP1-NAD(P)H-CoQ10 Pathway

The FSP1-NAD(P)H-CoQ10 pathway is considered to be a pathway that can regulate lipid peroxidation and ferroptosis together with the GPX4 pathway. Bersuker et al. used a synthetic lethal CRISPR/Cas9 screen to identify apoptosis-inducing factor mitochondrial 2 (AIFM2) as a ferroptosis resistance factor, which is now named ferroptosis suppressor protein 1 (FSP1) (Bersuker et al., 2019). FSP1 uses NAD(P)H to catalyze the regeneration of CoQ10, preventing lipid peroxidation and inhibiting ferroptosis.

## The Link Between Ferroptosis and Cancer

Evidence that has emerged in recent years suggests that ferroptosis may be an adaptive process that is key to eradicating the carcinogenic cells. Due to different necessary metabolic states, different cell lines have various sensitivities to ferroptosis. **Table 4** summarizes some known tumor cells that are sensitive to ferroptosis. Next, we discuss the connection between several specific types of cancer cells and ferroptosis.

**Table 4:** Tumor cells sensitive to ferroptosis.

Cancer type	Therapeutic	Type of evidence	Reference
Hepatocellular carcinoma	Erastin	Cell culture	(Dixon et al., 2012)
	Sorafenib	Cell culture; Cancer cells from patients	(Lachaier et al., 2014)
	Solasonine	Cell culture; Mice xenotransplantation model	(Jin et al., 2020)
Gastric carcinoma	Erastin	Cell culture	(Wang et al., 2016)
	Sulfasalazine(SAS)	Cell culture	(Okazaki et al., 2018)
	Buthionine sulfoximine (BSO)	Cell culture	(Wang et al., 2016)
Ovarian carcinoma	Erastin	Cell culture	(Zhou et al., 2019)
	Artesunate	Cancer cells from patients	(Greenshields et al., 2017)
Pancreatic carcinoma	Erastin	Cell culture	(Ohman et al., 2016)
	Artesunate	Cell culture	(Eling et al., 2015)
	Sorafenib	Cell culture	(Lachaier et al., 2014)
	Piperlongumine	Cell culture	(Yuki et al., 2018)
Breast carcinoma	Erastin	Cell culture	(Ma S. et al., 2017)
	Siramesine	Cell culture	(Ma S. et al., 2017)
	Lapatinib	Cell culture	(Ma S. et al., 2017)
	Sulfasalazine(SAS)	Cell culture	(Yu et al., 2019)
Colorectal carcinoma	Erastin	Cell culture; Mice xenotransplantation model	(Jiang et al., 2015b)
	Sorafenib	Cell culture	(Lachaier et al., 2014)
Melanoma	BAY 87-2243	Cell culture; Mice xenotransplantation model	(Schöckel et al., 2015)
	Sorafenib	Cell culture; Mice xenotransplantation model	(Lachaier et al., 2014)
Head and neck cancer	Artesunate	Cell culture; Mice xenotransplantation model	(Roh et al., 2017)
Kidney carcinoma	Erastin	Cell culture; Mice xenotransplantation model	(Jiang et al., 2015b)
	Sorafenib	Cell culture	(Lachaier et al., 2014)
Lung carcinoma	Sorafenib	Cell culture	(Lachaier et al., 2014)
Glioblastoma	Temozolomide	Cell culture; Mice xenotransplantation model	(Buccarelli et al., 2018)
Lymphoma	Sulfasalazine (SAS)	Cell culture; Mice xenotransplantation model	(Gout et al., 2001)
Retinoblastoma	Sorafenib	Cell culture; Mice xenotransplantation model	(Louandre et al., 2015; Krainz et al., 2016)

### Hepatocellular Carcinoma 

The current clinical treatment of hepatocellular carcinoma (HCC), especially advanced liver cancer, is not very satisfactory. Sorafenib has been approved by the Food and Drug Administration (FDA) for the treatment of hepatocellular carcinoma. Induction of ferroptosis in hepatocellular carcinoma is a significant way for sorafenib to play a role. The p62-Keap1-Nrf2 pathway (**Figure 1**) has an excellent effect on ferroptosis in liver cancer cells. Substrate adaptor p62 (also called sequestosome 1) protein inhibits the degradation of Nrf2 by inactivating the Keap1 protein. Sun et al. found that Erastin and sorafenib-treated HCC cell lines exhibited inhibitory growth under Nrf2 inhibition, where Nrf2 is a negative ferroptosis regulator (Sun et al., 2016a). When sorafenib treated HCC cells, the expression level of retinoblastoma (RB) protein decreased, and the cell death rate increased by 2–3 times compared with the cells that generally expressed RB protein. Hence, HCC cells with low RB protein levels are more sensitive to ferroptosis (Louandre et al., 2015). In response to the above findings, the application of ferroptosis to the treatment of hepatocellular carcinoma has good clinical application prospects, and the treatment of RB protein targeting will also become the direction of future research.

### Gastric Carcinoma 

Gastric carcinoma (GC), the fourth most common malignant tumor all over the world, has a death rate of 80% in more than 70% of countries (Yao and Kong, 2020). Hao et al. found that silencing human cysteine dioxygenase 1 (CDO1) led to the inhibition of Erastin-induced ferroptosis in GC cells. GPX4 and CDO1 expression can be regulated by MYB proto-oncogene transcription factor and the CDO1 promoter during ferroptosis. CDO1 can convert cysteine to taurine, reduce the availability of cysteine, and can also limit glutathione synthesis to inhibit the antioxidant capacity of cells, triggering ferroptosis (Hao et al., 2017). Actinidia chinensis Planch (ACP) is an approved anti-tumor drug for clinical use. Gao et al. found that ACP may achieve anti-tumor effects by promoting ferroptosis, apoptosis and inhibiting mesenchymal phenotype (Gao et al., 2020).

### Ovarian Carcinoma

Ovarian carcinoma, the fifth crucial cause of cancer death among women, has a high recurrence rate. It is prone to chemical resistance and eventually develops into end-stage disease (Siegel et al., 2019). Basuli D et al. found that highly serous ovarian cancer (HGSOC) tumor tissue has strong iron absorption and retention capacity, which can be proved by an increase in transferrin receptor 1 (TFR1) or ferritin, a decrease in ferroportin. Besides, they observed a similar situation in genetic models of the tumor-initiating cells (TICs) of ovarian cancer (Basuli et al., 2017). The above biological processes can cause iron overload in cells, which lays the foundation for the occurrence of ferroptosis. In the experiments of Anna *et al.*, artesunate (ART) can induce ROS accumulation in ovarian cancer cells and ferroptosis (Greenshields et al., 2017). A recent study showed that Erastin can reverse the resistance of ovarian cancer cells to docetaxel by inhibiting the pump activity of ABCB1. The combined use of Erastin and docetaxel provides a new option for the treatment of ovarian cancer resistance (Zhou et al., 2019).

### Pancreatic Carcinoma

Pancreatic cancer is extremely lethal, with a survival rate of less than 5% over five years. It is prone to early metastasis, develops rapidly, and is resistant to standard therapies (Rao et al., 2019). Eling et al. used ferroptosis inhibitor ferrostatin-1 to prevent ART-induced cell death, suggesting that artesunate-induced Panc-1 cell death is caused by ferroptosis. Compared to wild-type *KRAS* BxPC-3 cells, ART can more effectively trigger the iron-dependent ferroptosis of the *KRA*S mutant PDAC cell line (Eling et al., 2015). *KRAS* mutations are common in PDAC (Lanfranca et al., 2019). *KRAS* can upregulate ROS by regulating mitochondrial respiration, TFRC, autophagy, or NADPH, etc. (Storz, 2017). This level of ROS can be maintained at a moderate level by the action of GPX4 or the antioxidant system. Once affected by ferroptosis inducers, ROS levels will rise to lethal levels. Wang et al. used quantitative real-time PCR to measure the mRNA level of GRP78 in the *KRAS* mutant PDAC cell line treated with ART and found that the expression of GRP78, an ER chaperone protein, was increased. Both *in vivo* and *in vitro* experiments demonstrated that stable knockdown of GRP78 could make ART-treated mutant cells more sensitive to ferroptosis (Eling et al., 2015). In the future, inhibit the expression of GRP78, a protein highly expressed in cancer cells, to resist drug-treated cancer cell ferroptosis resistance needs to be expanded to more cancers. ART may promote ferroptosis induction by modulating the expression of the iron-related genes, which contribute to ferroptosis. These findings provide a promising way for PDAC treatment.

### Breast Carcinoma

The incidence of breast carcinoma is high, plagued a large number of women. Triple-negative breast cancer (TNBC), with a poor prognosis and a high risk of early metastasis, is a more aggressive subtype of breast cancer (Bao and Prasad, 2019). TNBC currently lacks effective targeted treatment, with a poor prognosis, usually based on chemotherapy. Knocking down *CHAC1* can greatly inhibit cystine hunger and reduce the availability of cysteine to resist ferroptosis (Chen et al., 2017). In MDA-MB-231 cells treated by siramesine and lapatinib, transferrin was affected, iron metabolism-related pathways for ferroptosis were stimulated. At the same time, the level of SLC7A11 protein increased, indicating that the Xc-system also takes effect (Ma et al., 2016). MUC1-C is a transmembrane oncoprotein that is often overexpressed in breast cancer. CD44v (CD44 variant) is a marker of cancer stem-like cells (CSCs) that can stabilize xCT transporters. The interaction of MUC1-C, CD44v, and xCT can regulate the expression of GSH and affect ferroptosis in breast cancer cells (Hasegawa et al., 2016).

### Colorectal Carcinoma 

Colorectal carcinoma (CRC) is a common fatal disease. The incidence and mortality of CRC vary widely around the world. In addition to suppressing or promoting specific genes (*SLC7A11*, *GLS2*) Outside the expression, Xie et al. experimental results indicate that *TP53* inhibits dipeptidyl peptidase 4 (DPP4) activity in an independent way of transcription, which can restrain Erastin-induced ferroptosis in colorectal carcinoma (Xie et al., 2017). Ferroptosis, through regulation of tumor protein p53 has become a new research direction in the treatment of colorectal carcinoma. Chen et al. found β-elemene is a unique natural ferroptosis inducer. β-elemene and cetuximab, which were combined to treat *KRAS* mutant CRC cells, could induce ferroptosis (Chen et al., 2020). Shen et al. demonstrated that RB could inactivate GPX4 to trigger ferroptosis in CRC cells, which can inhibit the growth of CRC cells and tumor formation (Shen et al., 2020).

### Melanoma

In a study of melanoma, miR-137, a vital tumor inhibitory factor, could inhibit glutamine transporter SLC1A5 to modulate ferroptosis. And knockdown of miR-137 could strengthen ferroptosis induced by Erastin (Luo et al., 2018). The voltage-dependent anion channel (VDAC) on the outer mitochondrial membrane can control the entry and exit of ions. Yang et al. found that the downregulation of Nedd4 can prevent the degradation of VDAC2/3 protein induced by Erastin, thus finally preventing ferroptosis (Yang et al., 2020). A recent study proved that low-level laser irradiation and natural herbal ingredient gallic acid (GA) could inactivate GPX4 to trigger ferroptosis in A375 melanoma cancer cells (Khorsandi et al., 2020).

## Current Status of Research on Ferroptosis Treatment

### Ferroptosis-Inducing Therapy

#### RSL3 and Erastin

RSL3 and Erastin, which are inconformity with pharmacokinetic standards *in vivo* application for poor water solubility and unstable metabolism, are two frequently-used ferroptosis inducers. To meet the clinical demand, optimizing the small molecular framework of RSL3 and Erastin has become the research direction of some researchers (Yang et al., 2014). In the study of Erastin with Ras selective lethality, Yang et al. observed that the target of Erastin is not Ras, but mitochondrial VDAC, which induces cell death through the RAS-RAF-MEK pathway (Yang and Stockwell, 2008). A more stable form known so far is piperazine-coupled Erastin. In one approach, folate (FA)-labeled Erastin-loaded exosomes are used to form FA-erased (Erastin@ FA-exo)-vectored exosomes to target FA receptor Overexpressed TNBC cells (Yu et al., 2019).

#### Sorafenib

Lachaier et al. determined that sorafenib is an oncogenic kinase inhibitor and can be used as an inducer of ferroptosis in hepatocellular carcinoma (Lachaier et al., 2014) and the therapeutic drugs in advanced Renal cell carcinoma (RCC). Mechanistically, Sorafenib can inhibit Xc-system (Dixon et al., 2014). Sorafenib induced cell death in hepatocellular carcinoma cells was suppressed by ferropstatin-1 and iron-chelators. Louandre et al. reported that sorafenib could induce ferroptosis, and found that tumor-suppressive retinoblastoma protein 1 (RB1) inhibits ferroptosis caused by sorafenib treatment.

#### Sulfasalazine

Sulfasalazine is mainly used as an anti-inflammatory drug for the treatment of rheumatic polyarthritis and chronic ulcerative colitis. Tina Sehm's experiments demonstrated that sulfasalazine induces ferroptosis in glioma cells by inhibiting the Xc-system while reducing tumor edema and seizures (Sehm et al., 2016). However, Yamaguchi et al. found no ferroptosis in MEFs treated with sulfasalazine (Yuki et al., 2018). A reasonable explanation is that there are differences in the sensitivity of different cell lines to ferroptosis.

#### Artemisinin and Its Derivatives

Artemisinin and its derivatives, in addition to being used for anti-malaria, has also been shown to be useful for cancer treatment (Arjen et al., 2005). Artesunate can induce iron-dependent and oxidative stress ferroptosis in PDAC cell lines, which can be blocked by iron chelator deferoxamine or ferrostatin-1 (Eling et al., 2015). Roh et al. found that artesunate can induce GSH depletion and lipid peroxidation, and selectively induce ferroptosis in head and neck cancer (HNC) cells without damaging normal cells. They also found that compared with cisplatin-sensitive cell lines, the effect of artesunate to induce ferroptosis is not good in some cisplatin-resistant HNC cell lines, which is due to the activation of the Nrf2-antioxidant response element (ARE) signaling pathway. The combination of the Nrf2 inhibitor trigonelline (or the silence of Nrf2) and artesunate can effectively kill cisplatin-resistant HNC cells, which also provides a method for overcoming ferroptosis resistance (Roh et al., 2017). Du et al. found that dihydroartemisinin (DHA) can induce ferroptosis in acute myeloid leukemia (AML) cells through autophagy degradation of ferritin. This supports the development of artemisinin and its derivatives as possible therapeutic agents for AML (Du et al., 2019).

#### Others

Research on ferroptosis has gradually increased, and more compounds that induce ferroptosis have been found. **Table 5** summarizes some small molecules and drugs related to ferroptosis in the current study. In the experiments of Ma et al., Siramesine and lapatinib can be used synergistically to induce an increase in iron levels and trigger ferroptosis (Ma S. et al., 2017). In addition to some commonly used ferroptosis drugs, some natural compounds such as Vitamin E (Ma S. et al., 2017), Baicalein (Xie et al., 2016), β-elemene (Chen et al., 2020), gallic acid (Khorsandi et al., 2020) can regulate ferroptosis by affecting the level of lipid peroxidation.

**Table 5:** Small molecules and drugs related to ferroptosis.

Classification	Reagent	Signal target	Test cells	FDA Approved/Clinical Use	Reference
Ferroptosis inducer	Erastin and its analogs	System Xc-; VDAC2/3	HT-1080 cell; A-673 cell; Calu-1 cell; BJeLR cell, etc.	No	(Yagoda et al., 2007; Dixon et al., 2012)
	Sorafenib	System Xc-	HCC cell; HT-1080 cell; Calu-1 cell, etc.	Yes/Renal cell carcinoma and hepatocellular carcinoma treatment	(Louandre et al., 2013; Dixon et al., 2014; Louandre et al., 2015)
	Sulfasalazine(SAS)	System Xc-; GPX4	Nb2-SFJCD1 cell; HT-1080 cell; BJeLR cell, etc.	Yes/Ulcerative colitis and rheumatoid arthritis treatment	(Gout et al., 2001; Sehm et al., 2016)
	Artemisinin and its derivatives	GSH depletion; Increased cellular iron	PDAC cell lines	Yes/Malaria treatment	(Eling et al., 2015)
	Buthionine sulfoximine (BSO)	GSH depletion	Pfa1 cell; BJeLR cell, etc.	No/Clinical trial for neuroblastoma treatment	(Harris et al., 2015; Yang et al., 2014)
	Cisplatin (CDDP)	GSH depletion	H1299 cell; A549 cell; HCT116 cell; MEFs, etc.	Yes/Treatment of various cancers	(Guo et al., 2018; Sato et al., 2018)
	(1S, 3R)-RSL3	GPX4	HT-1080 cell; Calu-1 cell; BJeLR cell, etc.	No	(Hangauer et al., 2017; Dixon et al., 2012)
	DPI7	GPX4	KBM7 cell	No	(Dixon et al., 2015; Yang et al., 2014)
	FIN56	GPX4 and SQS	HT-1080 cell; BJeLR cell, etc.	No	(Shimada et al., 2016)
	FINO_2_	Iron oxidation; Indirect inhibition of GPX4	HT-1080 cell	No	(Gaschler et al., 2018)
	Statins	Block biosynthesis of CoQ10	HCC4006 cell	Yes/Hypolipidemic	(Viswanathan et al., 2017)
	Lapatinib	Iron transport blocker	MDA MB 231 cell; SkBr3 cell	Yes/Breast cancer treatment	(Ma et al., 2017a)
	Siramesine	Increased cellular iron	MDA MB 231 cell; SkBr3cell	Yes	(Ma et al., 2017a)
Ferroptosis inhibitor	Vitamin E	Antioxidants	Q7 cells	Yes/Prevent habitual abortion	(Hinman et al., 2018)
	Baicalein	Activate Nrf2 pathway	PANC1 cell; BxPc3 cell	Yes	(Xie et al., 2016)
	Deferoxamine mesylate	Intracellular iron	BJeLR cell	Yes/Severe thalassemia treatment	(Yang and Stockwell, 2008)

At present, one of the main methods used in tumor treatment is chemotherapy, which is mainly to inhibit the synthesis of DNA or RNA at different stages of tumor growth and spread to inhibit the abnormal proliferation of tumors. Tumor resistance to drugs is an inherent ability of tumor cells. By improving DNA repair capacity, reducing drug intake, increasing drug pumping and other mechanisms to produce drug resistance. It is known that tumor cells are threatened by excessively elevated lipid peroxidation levels during chemotherapy. Some tumor cells use "redox remodeling" to raise the antioxidant system to a high level to balance with the threat and obtain a chance of survival (Yang et al., 2016). The increased iron demand exhibited by tumor cells indicates that it may be susceptible to ferroptosis.

Epithelial-mesenchymal transition (EMT), the differentiation of epithelial cells into mesenchymal cells, is an indispensable physiological phenomenon in the process of cell development. EMT is very important in inducing tumor metastasis (Marcucci et al., 2016). It provides malignant tumor cells with a "barrier" to prevent death, and makes tumor cells resistant to multiple treatment options. EMT promotes the synthesis of PUFAs in tumor cells by activating zinc-finger E-box binding homeobox 1 (ZEB1). From the amino acid mechanism described previously, we know that the metabolism of PUFAs by GPX4 can protect cells from ferroptosis caused by lipid peroxidation. This undoubtedly exposed the weakness that the mesenchymal cells are GPX4-dependent. The dependence of GPX4 has been confirmed in various cancer cell lines with mesenchymal status (Behan et al., 2019). Ferroptosis inducers that can target GPX4 or regulate GSH levels have become a way to eliminate drug resistance in mesenchymal cancer cells (Viswanathan et al., 2017).

Ferroptosis can not only be used to eliminate the drug resistance of tumor cells in mesenchymal state, but also effectively suppress the acquired drug resistance of tumor cells. Recent studies have found that drug-tolerant persister cells that are still alive after multiple rounds of chemotherapy also have a dependence on GPX4 (Hangauer et al., 2017). It is hopeful that the inhibition of GPX4 can induce ferroptosis in persistent cells to prevent tumor recurrence.

Roh et al. have shown that inducing ferroptosis can reverse the cisplatin resistance of the HNC cells (Roh et al., 2016). From similar studies as shown in **Table 6**, it seems that inducing ferroptosis to overcome resistance to anticancer drugs is a very promising research direction and deserves a more in-depth study.

**Table 6:** Studys on overcoming tumor drug resistance by inducing ferroptosis.

Cancer type	Test cells	Tolerant drug	Use ferroptosis to overcome drug resistance	Reference
Colorectal cancer	HCT116 cell; HT29 cell; LOVO cell; DLD1 cell	Cisplatin	Sulfasalazine treatment can improve the anticancer activity of cisplatin	(Ma et al., 2015)
Head and neck cancer	AMC-HN3R cell; AMC-HN4R cell; AMC-HN9R cell	cisplatin	Erastin and sulfasalazine treatment can overcome resistance to cisplatin	(Roh et al., 2016; Roh et al., 2017)
Ovarian cancer	A2780 cell; A2780DDP cell	Cisplatin	Erastin and artesunate treatment can improve the anticancer activity of cisplatin	(Sato et al., 2018; Wang et al., 2015)
Ovarian cancer	A2780 cell; A2780/Taxol cell	Docetaxel	Erastin treatment can overcome resistance to docetaxel	(Zhou et al., 2019)
Non–small cell lung cancer	H3255 cell; PC9 cell; H1299 cell; A549 cell	Cisplatin	Erastin treatment can improve the anticancer activity of cisplatin	(Yamaguchi et al., 2013)
Gastric carcinoma	SC-M1 cell; AGS cell; AZ521 cell	Cisplatin	Erastin and sulfasalazine treatment can improve the anticancer activity of cisplatin	(Wang et al., 2016)
Acute myeloid leukemia	HL60 cell	Cytarabine, doxorubicin	Low-dose Erastin treatment can significantly increase the anticancer activity of cytarabine, doxorubicin	(Yu et al., 2015)
Glioblastoma multiforme	GBM-N15 cell	Temozolomide	Erastin and sulfasalazine treatment can improve the anticancer activity of temozolomide	(Chen et al. 2015)
Hepatocellular carcinoma	Huh7 cell, HepaG2 cell,Hep3b cell	Sorafenib	Inhibition of metallothionein (MT)-1G can increase sensitivity to sorafenib	(Sun et al., 2016b)

### Nanomaterials for Ferroptosis-Based Cancer Therapy

The iron dependence of ferroptosis has been generally recognized, and nanoparticle materials using related mechanisms are also being gradually developed. They were using iron-containing ultrafine particles material to release iron to the tumor site, triggering Fenton reaction to generate ROS to induce cell death. Yue et al. designed a new ferroptosis inducer FePt-PTTA-Eu^3+^-FA (FPEF), which can be used for anti-cancer and inhibit metastasis (Yue et al., 2018). Zhang et al. found that Sorafenib-modified iron-based nanoparticles are more effective at inhibiting proliferation and inducing the death of HepG2 cells *in vitro* than sorafenib alone (Zhang et al., 2013). Also, drugs can be loaded into iron-containing nanoparticles (NPs), which may start a more efficient effect. Ma et al. loaded cisplatin (IV) prodrugs into iron oxide nanoparticles (Ma P. et al., 2017). The released cisplatin can use GSH depletion and GPX4 inhibition to induce ferroptosis, and the released unstable iron ions catalyze the decomposition of H202 to produce toxic ·OH, causing cell death. Similar experiments have been reported, and it has become urgent to promote in-depth multi-disciplinary cooperation in this area.

## Summary and Outlook

In brief, ferroptosis is a newly discovered form of cell death in recent years. It is driven by lipid peroxidation and has iron dependence. The mechanism involved in ferroptosis is very complicated. In addition to the amino acid metabolism mechanism, lipid peroxidation metabolism mechanism and iron metabolism mechanism, various organelles and pathways are also involved. Research on the mechanism of ferroptosis still needs to be continuously enriched in order to provide more valuable methods for treating diseases. Although more and more researchers are devoted to studying the mechanism of ferroptosis and tumor therapy, there are still many problems remaining to be solved. The action mechanism of iron in ferroptosis is not yet precise. The mechanism by which iron works cannot be simply thought of as the accumulation of lipid ROS caused by the Fenton reaction. Whether other substances can be used as substitutes for iron is not yet known. Whether lipid peroxidation caused ferroptosis through the formation of micelles and pores is not entirely certain. The link between organelles and ferroptosis still needs more detailed research. Further research is still needed to make sure which cancer types are sensitive to ferroptosis in more detail. Moreover, although some experiments have found some proteins that play a regulatory role in ferroptosis, it is still expected to find specific markers for the occurrence of ferroptosis, creating new opportunities for tumor diagnosis and therapeutic intervention. The use of small molecules to induce ferroptosis to overcome chemotherapy resistance in tumor cells has become the focus of many researchers. We all believe that the development of drugs for ferroptosis in the future may become a boon for many kinds of patients, especially cancer patients.

## Author Contributions

XL drafted the manuscript by reviewing the literature. JP and YWe participated in the discussion and prepared the chart. The corresponding author YWu guided the formation of the entire manuscript. All authors contributed to the article and approved the submitted version.

## Funding

This work was supported by the Natural Science Foundation Project of Jiangsu Province (No.17KJB310001); Funding from Health and Health Commission of Jiangsu Province (LGY2018025); 333 project funding plan (BRA2019172); and College Students' scientific research projects(18AD0084).

## Conflict of Interest

The authors declare that the research was conducted in the absence of any commercial or financial relationships that could be construed as a potential conflict of interest.

## Abbreviations

ROS, reactive oxygen species; RCD, regulated cell death; NCCD, Nomenclature Committee on Cell Death; Glu, glutamate; GSH, glutathione; GPX4, glutathione peroxidases 4; PUFAs, polyunsaturated fatty acids; α-KG, α-ketoglutarate; TCA, tricarboxylic acid cycle; AOA, amino-oxyacetate; MEFs, mouse embryonic fibroblasts; SLC7A11, the glutamate/cystine antiporter solute carrier family 7 member 11; CBS, cystathionine β-synthase; GCLC, glutamate-cysteine ligase; GSS, glutathione synthetase; GR, glutathione reductase; RPE, retinal pigment epithelial; RSL3, Ras-selective lethal small molecule 3; FIN56, ferroptosis-inducing agents 56; LOXs, lipoxygenases; ACSL4, acyl-CoA synthetase long-chain family member 4; LPCAT3, lysophosphatidylcholine acyltransferase 3; MAPK, mitogen-activated protein kinase; 4-HNE, 4-hydroxynonenal; LIP, labile iron pool; NCOA4, nuclear receptor coactivator 4; DMT1, divalent metal transporter 1; BMSCs, bone marrow stromal cells; FH, fumarate hydratase; CISD1, CDGSH iron sulfur domain 1; CDDO, 2-amino-5-chloro-N, 3-dimethylbenzamide; HSP90, heat shock protein 90; CMA, chaperone-mediated autophagy; ER, endoplasmic reticulum; ATF4, activating transcription factor 4; CHAC1, cation transport regulator homolog 1; Nrf2, nuclear factor erythroid 2-related factor 2; MVA, mevalonate; IPP, isopentenyl pyrophosphate; HMGCR, HMG-CoA reductase ; SQS, squalene synthase; AIFM2, apoptosis-inducing factor mitochondrial 2; FSP1, ferroptosis suppressor protein 1; HCC, hepatocellular carcinoma; FDA, Food and Drug Administration; RB, retinoblastoma; GC, gastric carcinoma; CDO1, cysteine dioxygenase 1; ACP, actinidia chinensis planch; HGSOC, highly serous ovarian cancer; TFR1, transferrin receptor 1; TICs, tumor-initiating cells; PDAC, pancreatic ductal adenocarcinoma; PEITC, phenylethyl isothiocyanate; TNBC, triple-negative breast carcinoma; CSCs, cancer stem-like cells; ART, artesunate; DPP4, dipeptidyl peptidase 4; VDAC, voltage-dependent anion channel; GA, gallic acid; FIT, ferroptosis-inducing therapy; ARE, antioxidant response element; DHA, dihydroartemisinin; AML, acute myeloid leukemia; EMT, epithelial-mesenchymal transition; ZEB1, zinc-finger E-box binding homeobox 1; RCC, renal cell carcinoma; CDDP, cisplatin.
